# Piperacillin-Tazobactam (TZP) Resistance in *Escherichia coli* Due to Hyperproduction of TEM-1 β-Lactamase Mediated by the Promoter *Pa/Pb*

**DOI:** 10.3389/fmicb.2019.00833

**Published:** 2019-04-16

**Authors:** Kaixin Zhou, Ying Tao, Lizhong Han, Yuxing Ni, Jingyong Sun

**Affiliations:** Department of Clinical Microbiology, Ruijin Hospital, Shanghai Jiao Tong University School of Medicine, Shanghai, China

**Keywords:** TZP resistance, *Escherichia coli*, Pa/Pb, β-lactamase, antimicobial

## Abstract

TEM-1, mediated by plasmid and transposon, is the most commonly encountered β-lactamase in Gram-negative bacteria. Four different promoters upstream of *bla*_TEM_-related genes have been identified: the weak *P3* promoter, and the strong promoters *Pa/Pb*, *P4*, and *P5.* In this study, we investigated the genetic basis of a clinical strain of *Escherichia coli* (RJ904), which was found to be resistant to BLBLIs (β-lactam/β-lactamase inhibitors), including amoxicillin-clavulanate, ticarcillin-clavulanate (TCC), and piperacillin-tazobactam (TZP) but sensitive to third-generation cephalosporins. The conjugation test and S1-nuclease pulsed-field gel electrophoresis (S1-PFGE) demonstrated that transfer of this resistance was mediated by a ca. 100 kb plasmid. The transformant with TZP resistance was screened out with the shortgun cloning. Sequence analysis revealed that the recombinant plasmid contained a *bla*_TEM-1b_ gene with the strong promoter *Pa/Pb*. Different plasmids were cloned based on the clone vector pACYC184 with the insertion of the *bla*_TEM-1b_ gene with promoters *Pa/Pb* or *P3*. Susceptibility to TZP was determined by the *E*-test, agar dilution, and broth microdilution. The level of *bla*_TEM-1b_-specific transcription was determined by quantitative real-time PCR. Substitution of *Pa/Pb* for *P3* resulted in a 128-fold decline of the MIC value of TZP, from >1024 mg/L to 8 mg/L, and a significantly lower *bla*_TEM-1b_ expression level. Hyperproduction of TEM-1 β-lactamase mediated by the promoter *Pa/Pb* was responsible for high resistance to TZP in *E. coli*. Our data show possible risks of resistance development in association with the clinical use of TZP. The *bla*_TEM_ promoter modifications should be considered for whole genome whole-genome sequencing-inferred bacterial antimicrobial susceptibility testing.

## Introduction

The production of β-lactamases is the predominant cause of resistance to β-lactam antibiotics in Gram-negative bacteria (Bonnet, 2004), including the hyperproduction of plasmid-mediated TEM-1 β-lactamases, production of extended-spectrum beta-lactamases (ESBLs), plasmid-mediated AmpC enzymes (Caroff et al., 1999) and carbapenem-hydrolyzing β-lactamases (carbapenemases)(Wu et al., 1994; Jacoby and Munoz-Price, 2005). Combining β-lactam and a β-lactamase inhibitor (BLBLIs) was a common strategy to overcome resistance (Chaibi et al., 1999; Perez-Llarena and Bou, 2009). However, resistance to BLBLIs has also been regularly observed (Pérez-Moreno et al., 2010; Waltner-Toews et al., 2011).

TEM-1 was described in the early 1960s as the first plasmid-mediated β-lactamase in Gram-negative bacteria (Datta and Kontomichalou, 1965). Being plasmid and transposon-mediated has facilitated its spread to other species of bacteria and it is now the most commonly encountered β-lactamase in Gram-negative bacteria (Bradford, 2001). The subgroups were defined and designated a, b, and c for a given *bla*_TEM_ gene derivative, because of their relation to a certain number of nucleotide differences in their structural gene sequence (Leflon-Guibout et al., 2000). The corresponding *bla*_TEM-1b_ gene derives from *bla*_TEM-1a_ by three base pair changes: C226T, C436T, and G604T, silent base pair change. *bla*_TEM-1c_ gene differs from *bla*_TEM-1a_ by the nucleotide substitution C436T, which is also silent. *bla*_TEM-2_ differs from *bla*_TEM-1a_ at position 317, where a A-to-C substitution leads to Gln39Lys (Goussard and Goussard, 1991). Previous studies identified four *bla*_TEM_ promoters: the weak *P3* promoter, and the strong promoters *Pa/Pb*, *P4*, and *P5* (Lartigue et al., 2002). *P3* corresponds to the promoter of the *bla*_TEM_ gene located in a Tn*2* or Tn*3* transposon (Sutcliffe, 1978; Lartigue et al., 2002; Partridge and Hall, 2005). A single-base pair mutation (C32T) results in the stronger overlapping promoters *Pa/Pb*, first found upstream of the gene *bla*_TEM-2_, and produces larger amounts of the enzyme compared with the promoter *P3* (Chen and Clowes, 1987a,b). Thus, an updated *bla*_TEM_ gene nomenclature was proposed on the basis of the sequences of structural *bla*_TEM_ genes and their promoters (Goussard and Courvalin, 1999).

Lartigue et al. (2002) assessed and compared the respective impact of the four promoters on β-lactam resistance. Among the recombinant plasmids, one with a *bla*_TEM-1b_ gene driven by a *Pa/Pb* promoter resulted in resistance to AMC and ticarcillin-clavulanate (TCC), but susceptibility to piperacillin-tazobactam (TZP) with a MIC value of 2 mg/L. In this study, the mechanism of TZP resistance was investigated in *Escherichia coli* RJ904, a clinical isolate containing the *bla*_TEM-1b_ gene with a *Pa/Pb* promoter. Experimental and genomic data support a role for *Pa/Pb* promoter regulation, leading to *bla*_TEM-1b_ hyperproduction, as the primary basis for TZP resistance in this isolate.

## Materials and Methods

### Ethics Statement

This study was approved by the ethics committee of Ruijin Hospital, School of Medicine, Shanghai Jiao Tong University, Shanghai, China and the Review Board exempted the requirement for written informed consent because this retrospective study only focused on bacteria and did not affect the patients.

### Bacterial Strains and Growth Condition

The clinical strain *E. coli* RJ904 was obtained from the blood specimen of a hospitalized patient in Shanghai, China (Ruijin Hospital, School of Medicine, Shanghai Jiao Tong University) in 2005. Ceftazidime was used for the medication. The patient’s condition improved after the treatment and the patient was discharged. The isolate was identified using VITEK2 automated systems (BioMérieux, France). All of the plasmids used in this study are listed in Supplementary Table S1. All cloning procedures were carried out in *E. coli* (DH5α), and antibiotics were used with suitable concentrations for plasmid selection when necessary. All the *E. coli* strains were routinely grown in Luria-Bertani (LB) broth (Oxoid) and incubated overnight at 35°C.

### Antimicrobial Susceptibility Testing

Susceptibility testing of all the antibiotics for the clinical strain RJ904, transconjugant RJ904C, and recombinant vectors RJ904-PA/PB was determined using the *E*-test (bioMérieux, France). The antibiotic susceptibility of the strains to piperacillin with a fixed concentration of tazobactam (TZP, 4 mg/L) was determined using three methods: *E*-test, agar dilution, and broth microdilution method. The results were interpreted based on the guidelines of the CLSI (2014).

### Conjugal Transfer Experiments and S1-Nuclease Pulsed-Field Gel Electrophoresis (S1-PFGE)

Conjugal transfer experiments were performed in broth culture using the strain RJ904 as the donor and the sodium azide-resistant strain *E. coli* J53Azi^r^ as the recipient. Selection was performed with piperacillin (100 mg/L), tazobactam (4 mg/L), and sodium azide (100 mg/L). The plasmid DNA of RJ904 and its transconjugant RJ904C was examined using S1-PFGE as previously described (Barton et al., 1995).

### Plasmid Construction

The principle features of all plasmids are listed in Supplementary Table S1.

First, the fragment of *bla*_TEM-1b_ gene was screened by the shortgun cloning. In brief, plasmid DNAs of pRJ904 were extracted with the Plasmid DNA Mini Kit (Omega). pRJ904 and the clone vector pACYC184 were digested with restriction enzymes *BamH*I and *Hind*III (Thermo Fisher Scientific) and ligated to construct a DNA library, which was used to transform the competent cells. Selection was then performed with piperacillin (100 mg/L), tazobactam (4 mg/L), and chloramphenicol (50 mg/L). The new cloned plasmid was named pRJ904-PA/PB.

The recombinant vector was cloned as described by Lartigue et al. (2002) using the same primers (BamHI-P-F and BamHI-P-R), clone vector, and restriction enzyme digestion site. pRJ904-PA/PB and p749 (MH491004) served as templates, respectively. p749 was a plasmid from *E. coli* retained by our laboratory that contained the *bla*_TEM-1b_ gene and promoter region with 99% base pair identity to pRJ904, except a point mutation (T32C) in the promoter region of *bla*_TEM-1b_, resulting in substitution of the promoter *Pa/Pb* for *P3*. The PCR products were purified and digested with *BamH*I (Thermo Fisher Scientific) and cloned into pACYC184 to construct plasmids pRJ904-PA/PB-P and pRJ904-P3-P. Both plasmids were cloned based on pACYC184, and the *bla*_TEM-1b_ gene was inserted; however, pRJ904-PA/PB-P contained the *Pa/Pb* promoter while pRJ904-P3-P contained the *P3* promoter.

After cloning, all of the plasmids were transformed into *E. coli* DH5α cells by using standard techniques (Denman, 1983). Selection was performed on an LB agar plate containing ampicillin (100 mg/L) and chloramphenicol (50 mg/L). Proper integration of all the constructs were verified by PCR amplification with the primers 184-F and 184-R binding on pACYC184, followed by sequencing of the PCR product. The direction of the *bla*_TEM-1b_ fragments in all the constructs were opposite to the *tetR* gene of pACYC184 in order to rule out the possible expression of the *tetR* gene.

### Transcriptional Analysis of *bla*_TEM-1b_

For real-time PCR, the indicated *E. coli* strains were grown in LB broth and harvested at an OD600 of 1. The RNA was extracted using RNeasy Mini Kit (Qiagen), and then used to generate cDNA with PrimeScript^TM^ RT Master Mix (TaKaRa). RT-PCR was performed using SYBR green PCR master mix (Applied Biosystems) with the primer pair TEM-F and TEM-R (Supplementary Table S2) on a cobas z480^®^ system (Roche) (Her and Schutzbank, 2018). Amplification of the 16S rRNA gene (as an endogenous control) was performed to standardize the amount of sample RNA or DNA added to a reaction. Relative quantification was determined by the 2^-ΔΔCT^ method. Each assay was performed in triplicate with three independent cultures. Statistical comparisons were performed by one-way analysis of variance (ANOVA) followed by Holm-Sidak tests to compare selected data pairs. Values of *P* < 0.05 were considered statistically significant.

### Nucleotide Sequence Accession Number

The nucleotide sequence containing a *bla*_TEM-1b_ gene with the promoter *Pa/Pb* from the clinical strain RJ904 has been deposited in the GenBank sequence database under accession number MH357372.

## Results

### Plasmid-Mediated Transfer of the Resistance to β-Lactam and β-Lactamase Inhibitor Combinations

The clinical isolate RJ904 was determined by *E*-test and found to be highly resistant to BLBLIs, including AMC, TCC, and TZP (MICs>256 mg/L), but was susceptible to third-generation (Table 1). Resistance to TZP was transferable using the broth mate conjugation assay. Although the transconjugant RJ904C showed a decreased MIC to third-generation cephalosporins, the MIC values of BLs and BLBLIs were all significantly higher than that of the recipient strain *E. coli* J53Azi^r^. The results of S1-PFGE confirmed the presence of a ca. 100 kb plasmid in both the donor strain RJ904 and the transconjugant RJ904C (Supplementary Figure S1).

**Table 1:** Antibiotic susceptibilities of *E. coli* strains RJ904, RJ904C, RJ904-PA/PB, RJ904-P3.

Antibiotics	MIC (mg/L)				
	J53	DH5α	RJ904	RJ904C	RJ904-PA/PB
Amoxicillin	4	4	>256	>256	>256
Piperacillin	2	2	>256	>256	>256
Amoxicillin-clavulanate	4	2	>256	>256	>256
Ticarcillin-clavulanate	2	1	>256	>256	>256
Piperacillin-tazobactam	1	0.5	>256	>256	>256
Cefazolin	4	4	>256	>256	>256
Cefuroxime	4	4	32	4	8
Cefoperazone	0.125	0.064	>256	32	256
Cefotaxime	0.032	0.032	0.5	0.064	0.25
Ceftazidime	0.125	0.125	2	0.5	2
Cefoxitin	4	4	64	4	4


### Hyperproduction of TEM-1b β-Lactamase Mediated by the Promoter *Pa/Pb*

**FIGURE 1:**
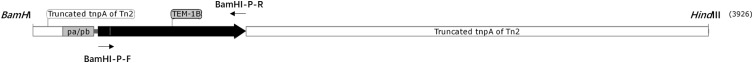
Schematic representation of the 3.9-kb *BamH*I and *Hind*III-digested embedded TEM-1b fragment (black arrow) and the promoter *Pa/Pb* (white rectangle). Truncated *tnpA* of Tn2 was placed on both sides of the resistance gene (gray). The sites for primers BamHI-P-F and BamHI-P-R for PCR clone are also indicated.

The shortgun cloning and sequence analysis revealed that the recombinant vector pRJ904-PA/PB contained a DNA insertion of approximately 3.9 kb containing the *bla*_TEM-1b_ gene, located on the resolvase gene (*tnpR*) of Tn2, and the promoter upstream the *bla*_TEM-1b_ gene was *Pa/Pb* (Figure 1). The MIC value of BLs and BLBLIs of *E. coli* RJ904-PA/PB was similar to that of the transconjugant RJ904C (Table 1).

**FIGURE 2:**
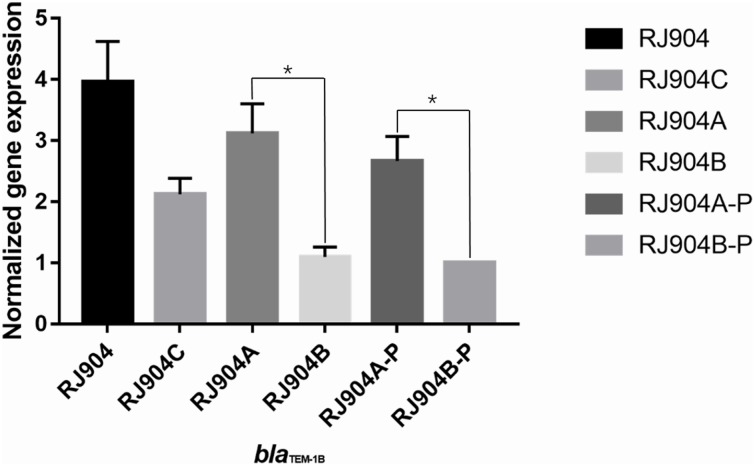
Relative *bla*_TEM-1b_ expression levels of all the strains. ^∗^*p* < 0.01. RJ904-P3-P served as a reference. RJ904-PA/PB and RJ904-PA/PB-P also demonstrated a significantly higher *bla*_TEM-1b_ expression level than RJ904-P3-P.

The level of *bla*_TEM-1b_-specific transcription was determined by quantitative RT-PCR. As shown in Figure 2, RJ904-PA/PB demonstrated a significantly higher relative *bla*_TEM-1b_ expression level than RJ904-P3-P (*P* < 0.01).

### Expression of TEM-1b for pRJ904-PA/PB-P and pRJ904-P3-P

To further confirm that the resistance to TZP is caused by the promoter *Pa/Pb* and for comparison with the results of Lartigue et al. (2002), the plasmids pRJ904-PA/PB-P and pRJ904-P3-P were constructed.

The MIC value of TZP for all strains was determined by three different methods (Table 2). The MIC values of RJ904-PA/PB and RJ904-PA/PB-P were >256 mg/L in the *E*-test and were ≥024 mg/L in agar dilution and broth microdilution tests, indicating no difference from the susceptibility profile of the original strain RJ904 and the transconjugant RJ904C. However, RJ904-P3-P demonstrated significantly declined MIC values of 8 mg/L (agar dilution and *E*-test) or 16 mg/L (broth microdilution test), and 4 mg/L (agar dilution and *E*-test) or 8 mg/L (broth microdilution test), respectively. Consistently, RJ904-PA/PB-P demonstrated a significantly higher *bla*_TEM-1b_ expression level than RJ904-P3-P.

**Table 2:** Susceptibility testing results of *E. coli* strains to piperacillin with 4 mg/L of tazobactam (TZP).

Strain	*E*-test (mg/L)^a^	Agar dilution (mg/L)	Broth microdilution (mg/L)
ATCC25922	2	2	1
J53	1	1	1
DH5α	0.5	1	2
RJ904	>256	≥1024	≥1024
RJ904C	>256	≥1024	≥1024
RJ904-PA/PB	>256	≥1024	≥1024
RJ904-PA/PB-P	>256	≥1024	≥1024
RJ904-P3-P	4	4	8


*^*a*^MIC breakpoint (mg/L): *S* ≤ 16/4; I: 32/4–64/4; *R* ≥ 128/4 (CLSI).*

## Discussion

The conjugation experiment demonstrated that resistance to TZP can be transferred from RJ904 to J53Azi^r^. The short gun method was used to screen out a strain that was highly resistant to TZP, and sequence analysis revealed that the plasmid harbored a 3.9-kb insertion embedded in the *bla*_TEM-1b_ gene with the strong promoter *Pa/Pb*. The mutant strain RJ904-P3-P with the weak promoter *P3* demonstrated substantially declining MIC values to TZP. Moreover, RJ904-PA/PB and RJ904-PA/PB-P demonstrated a higher *bla*_TEM-1b_ expression level than RJ904-P3-P. Altogether, these data provide strong functional evidence that the acquisition of TZP resistance was due to the hyperproduction of TEM-1b β-lactamases mediated by the strong promoter *Pa/Pb*.

Lartigue et al. (2002) suggested that the *bla*_TEM-1b_ gene with a *Pa/Pb* promoter could contribute to the resistance to AMC and TCC but not to TZP with a MIC value of 2 mg/L, suggesting the potential importance of this promoter for β-lactam resistance. However, we found that strain RJ904-PA/PB, which also contained the *bla*_TEM-1b_ gene with a *Pa/Pb* promoter, was highly resistant to TZP with a MIC value >256 mg/L. To identify possible causes of the difference, we replicated the experiment of Lartigue et al. (2002) using the exact same primers, clone vector, and restriction enzyme digestion site to clone the plasmid with the *bla*_TEM-1b_ gene and *Pa/Pb* promoter (pRJ904-PA/PB-P), which was compared to a plasmid with the *P3* promoter (pRJ904-P3-P). We next determined the MIC value of TZP of all strains. Since several authors have claimed that the MIC determination of TZP can be method-dependent and strains exhibited discordant behavior and heterogeneous resistance in different methods (Creely et al., 2013; Shubert et al., 2014), we used three methods for susceptibility testing to avoid the methodological impact: broth microdilution, agar dilution, and *E*-test. Several studies have compared the results of TZP susceptibility testing with broth microdilution and agar dilution methods for isolates of various species,(Thomson et al., 2008; Creely et al., 2013; Steensels et al., 2013; Shubert et al., 2014) and broth microdilution showed a tendency toward higher MIC values than agar dilution (Steensels et al., 2013). In the present study, there was no difference in the MIC values of RJ904-PA/PB-P to those of strains RJ904, RJ904C, and RJ904-PA/PB regardless of the method used. All these strains with a promoter *Pa/Pb* demonstrated high resistance to TZP unlike Lartigue’s transformants, while strains with a promoter *P3* (RJ904-P3 and RJ904-P3-P) demonstrated a significantly declined MIC value ultimately becoming susceptible to TZP, which is consistent with the findings of Lartigue’s transformants with a *P3* promoter. *E. coli* DH5α was used as the recipient rather than *E. coli* NM554. However, RJ904, the transconjugant RJ904C (*E. coli* J53), and RJ904-PA/PB-P (*E. coli* DH5α) all demonstrated high resistance to TZP. These results indicate that the recipient will not have a great impact on the expression of drug-resistant genes.

Nevertheless, when we repeated the experiment, we reached a different conclusion. The strains with promoter *Pa/Pb* in our study demonstrated high resistance to TZP while Lartigue’s transformants was susceptible to TZP. Although the reason for this discrepancy is not yet clear, our results from several independent assessments all indicate that the resistance to TZP was due to hyperproduction of TEM-1b β-lactamases mediated by the strong promoter *Pa/Pb*. However, overexpression of *bla*TEM-1 can lead to resistance, including clavulanate and sulbactam (Stapleton et al., 1995; Waltner-Toews et al., 2011). *bla*_TEM-1_ hyperproduction resulting from an increase in *bla*_TEM-1_ gene dosage has also been documented (Wu et al., 1995; Waltner-Toews et al., 2011). Schechter et al. (2018) claimed that tandem *bla*_TEM-1_ gene amplification, leading to *bla*_TEM-1_ hyperproduction, as the primary basis for TZP resistance in *E. coli*. These results indicated that *bla*_TEM-1_ hyperproduction can lead to BLBLIs resistance, including TZP.

Whole-genome sequencing (WGS) can help to infer antimicrobial susceptibility accurately using a single assay (Ellington et al., 2017). However, most existing databases focus only on the commonly known resistance loci while neglecting the role of promoters. Our finding should be considered for the acquisition of more accurate WGS-inferred bacterial antimicrobial susceptibility testing. Importantly, these data add to the growing body of evidence that the same resistance gene with different promoters will result in completely different susceptibility testing results. Thus, when performing WGS-inferred AST, we should not only assess the resistance genes but should also analyze their promoter sequences simultaneously. Our finding also shed light on the possibility of a fast identification using a simple PCR and sequencing to identify strong promoters and weak promoters and to infer antimicrobial susceptibility.

## Author Contributions

All authors listed have made a substantial, direct and intellectual contribution to the work, and approved it for publication.

## Conflict of Interest Statement

The authors declare that the research was conducted in the absence of any commercial or financial relationships that could be construed as a potential conflict of interest.
