# Supplementary material for: Piperacillin-Tazobactam (TZP) Resistance in Escherichia coli Due to Hyperproduction of TEM-1 β-Lactamase Mediated by the Promoter Pa/Pb
Source: Front Microbiol. 2019 Apr 16;10:833. doi: 10.3389/fmicb.2019.00833 (PMC6476967; doi:10.3389/fmicb.2019.00833)
Supplement: Supplementary file 1 [file Table_1.docx]

Supplementary materials

Figure S1:


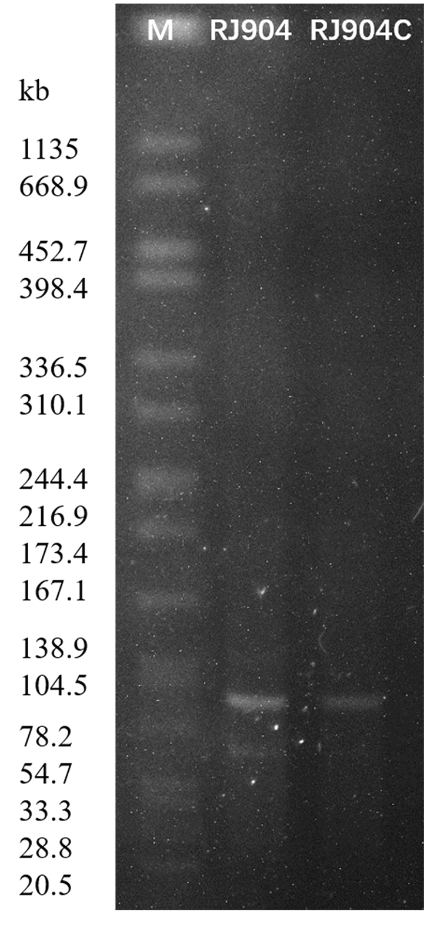


Pulsed-field gel electrophoresis (PFGE) profiles of *E. coli* RJ904 and its transconjugant, RJ904C. PFGE analysis showed the presence of a ca. 100 Kb plasmid in both the donor strain RJ904 and the transconjugant RJ904C. M: *Salmonella enterica* serotype Braenderup H9812 was digested with *Xba*I and used as a molecular size marker.

Table S1. Plasmids used in this study.

| Plasmid | Relevant genotype and characteristics | Reference or source |
| --- | --- | --- |
| pACYC184 | *E. coli* plasmid vector, Cm^r^ Tc^r^ | (Rose, 1988) |
| pRJ904 | Plasmid from RJ904 | This study |
| pRJ904-PA/PB | pACYC184 with restriction fragment (containing *bla_TEM-1B_* and promoter *Pa/Pb*) of RJ904 | This study |
| pRJ904-PA/PB-P | pACYC184 with promoter *Pa/Pb* and *bla_TEM-1B_* | This study |
| pRJ904-P3-P | pACYC184 with promoter *P3* and *bla_TEM-1B_* | This study |

| Primers | Sequence（5’→3’） | Reference or source |
| --- | --- | --- |
| Primers for plasmids construction |  |  |
| BamHI-P-F | CGCGGATCCATAAAATTCTTGAAGAC | This study |
| BamHI-P-R | CGCGGATCCTTACCAATGCTTAATCA | This study |
| Primers for pACYC184 |  |  |
| 184-F | GTCACTATGGCGTGCTGCTA | This study |
| 184-R | CGGTGATGTCGGCGATATAGG | This study |
| Primers for RT-PCR |  |  |
| 16S-F | GCATAACGTCGCAAGACCAAAG | (Xu et al., 2007) |
| 16S-R | TTCTTCATACACGCGGCATGG | (Xu et al., 2007) |
| TEM-F | ACCCAGAAACGCTGGTGAAA | This study |
| TEM-R | CGGGATAATACCGCACCACA | This study |

Table S2. Primers used in this study.

Rose, R.E. (1988). The nucleotide sequence of pACYC184. *Nucleic Acids Res* 16(1)**,** 355.

Xu, X., Wu, S., Ye, X., Liu, Y., Shi, W., Zhang, Y., et al. (2007). Prevalence and expression of the plasmid-mediated quinolone resistance determinant qnrA1. *Antimicrob Agents Chemother* 51(11)**,** 4105-4110. doi: 10.1128/AAC.00616-07.
